# Affects de la mère pendant la grossesse, relation mère-bébé, santé et développement du nourrisson à Kinshasa

**DOI:** 10.11604/pamj.2020.36.203.18294

**Published:** 2020-07-22

**Authors:** Daniel Okitundu Luwa E-Andjafono, Brigitte Imbula Essam, Adelin N´situ Mankubu, Ally Ndjukendi Omba, Timothée Kamanga Mbuyi

**Affiliations:** 1 Unité de Neuropsychologie, Département de Neurologie, Centre Neuro-Psycho-Psychopathologique, Université de Kinshasa, Kinshasa, République Démocratique du Congo,; 2 Département de Psychiatrie, Centre Neuro-Psycho-Psychopathologique, Université de Kinshasa, Kinshasa, République Démocratique du Congo,; 3 Département Psychologie, Section de Psychologie Clinique, Université de Kinshasa, Kinshasa, République Démocratique du Congo

**Keywords:** Affects de la femme enceinte, comportement du bébé, dépression maternelle, grossesse non désirée, Depression, body feelings, unintended pregnancy, infant´s health

## Abstract

**Introduction:**

le rôle de l´affectivité maternelle prénatale dans le développement du nourrisson est peu documenté dans notre milieu. L´objectif de cette étude était de déterminer les liens entre les sentiments de la mère à la connaissance de la grossesse, la peur de l´accouchement, la relation mère-bébé et le développement du nourrisson.

**Méthodes:**

en consultations préscolaires à Kinshasa, 120 mères âgées de 28,4 ans (± 12,18 ans), portant des bébés de 1 à 10 mois, ont participé à une étude transversale, par entretien et questionnaire, sur la relation mère-bébé, le comportement, la santé et le développement du bébé et l´affectivité maternelle. Les échelles de dépression maternelle postnatale d´Edimbourg (l´EPDS), de dépression et anxiété de Goldberg et les critères DSM-IV de dépression ont été utilisées. Il y a eu recours aux tests de Khi-carré et Mann-Whitney dans l´analyse statistique.

**Résultats:**

l´absence de joie relative à la grossesse était associée à la grossesse non désirée (p<0,001), l´absence du soutien du père de l´enfant (p=0,011), l´ennui au portage du bébé frustré (p=0,046), la conso labilité difficile du bébé (p<0,001), le comportement non affiliatif du bébé né d´une grossesse désirée (p=0 ,034), la santé infantile moins bonne (p=0 ,010) et la dépression maternelle (EPDS) (p=0,043). La peur de l´accouchement était associée au déficit de réponses aux signaux du bébé (p=0 ,002) et la tension au portage du bébé frustré (p=0,02).

**Conclusion:**

les affects négatifs pendant la grossesse prédiraient des troubles de la relation mère-bébé, de la santé et du développement du nourrisson.

## Abstract (en)

**Introduction:**

the impact of prenatal maternal affectivity on infant development is poorly documented in Africa. The purpose of our study is to determine the relationship between mother feelings about pregnancy and childbirth, infant´s behavior and development.

**Methods:**

one hundred and twenty mothers aged 28,4 years (± 12,18 years) with their babies aged 38,70 months (± 19,19) were enrolled in a cross-sectional study based on an interview and questionnaire on maternal affectivity, mother-child relationship, infant´s behavior and psychosocial development. Maternal depression was assessed by Edinburgh (EPDS), Goldberg Depression and anxiety scales and DSM-IV (MINI) criteria. Chi-square and Mann-Whitney were used to describe the relationship between maternal affectivity and infant’s behavior and health.

**Results:**

unintended pregnancy and fear of childbirth were associated with high anxiety and depression scores. Negative feelings about pregnancy were associated with the lack of social support by the child´s father (0.0110), stress when women were pregnant with a frustrated child (p=0.046), difficult consolability (p<0.001), poor baby’s health (p=0.010), infant non-affiliative behavior (p=0.034) and depression identified by using EPDS (p=0.028). Fear of childbirth was associated with delayed or absent responses to infant´s signals (p=0.002) and stress if they were carrying a frustrated baby (p=0.020).

**Conclusion:**

negative feelings during pregnancy are predictive of maternal depression, mother-child relationship disorders, infant´s health and development.

## Introduction

Les conséquences du stress pendant la grossesse sur le développement de l´enfant ont fait l´objet de recherches soutenant l´hypothèse d´effets prolongés toute la vie [1-4]. Des études épidémiologiques autant que cliniques sont nécessaires pour en tirer des applications dans le traitement et la prévention des problèmes de santé et de développement en soins de santé primaires. Le bébé, partenaire relationnel de la mère, signifie aussi nourrisson désignant l´enfant depuis sa vie intra-utérine jusqu´à la fin de la 3^e^année [5]. Le bébé est en interaction avec sa mère dès le début de la grossesse par les désirs de la grossesse et de l´enfant ainsi que par les remaniements psychiques occasionnés par l´état de grossesse. Le stress maternel prénatal, comme en cas de grossesse non désirée, a des répercussions négatives sur le développement moteur et l´évolution comportementale du bébé [4, 6]. La dépression maternelle postnatale, qui peut être la continuité de la dépression maternelle de la grossesse [7], a des répercussions négatives sur le développement moteur, social et cognitif et la santé du bébé [8]. Les enfants de mères déprimées ont un tempérament difficile marqué par des pleurs excessifs et une faible conso labilité dès les premières heures après la naissance, ce qui amena Field à poser l´hypothèse d´une transmission prénatale des mécanismes de la détresse psychologique chez le bébé [9].

Une prévention précoce, basée sur une connaissance des facteurs psychosociaux influençant dès le début de la grossesse la relation mère-bébé, l´affectivité maternelle, la santé et le développement du bébé, est essentielle pour la protection de la santé maternelle et infantile (PMI). L´hypothèse de la présente recherche était que l´interaction interpersonnelle dès la conception détermine l´affectivité maternelle et le développement neurologique et psychosocial du bébé. Les sentiments de la future mère à la connaissance de sa grossesse et la peur de l´accouchement affectent l´interaction mère-bébé, l´affectivité maternelle et le développement du bébé. L´objectif général était de déterminer les liens entre l´affectivité maternelle pendant la grossesse et par rapport à l´accouchement, la relation mère-bébé, la santé et le développement du bébé dans la première année de la vie. Les objectifs spécifiques étaient les suivants: a) identifier les affects des mères dans notre milieu en rapport avec la grossesse, l´accouchement et la relation mère-bébé pendant la première année de la vie; b) déterminer les facteurs d´interaction interpersonnelle associés aux troubles affectifs maternels; c) déterminer le profil comportemental et sanitaire de bébés dans la première année en lien avec les affects de la mère relatifs à la connaissance de la grossesse et à l´accouchement.

## Méthodes

**Sujets:** l´étude a été réalisée auprès de 120 femmes consentantes entre septembre 2008 et février 2009 en consultations préscolaires dans un centre hospitalier de Kinshasa desservant une population en conditions de vie difficiles à l´est de la ville. Les mères avaient répondu à ces critères d´inclusion: être mère biologique d´un bébé âgé de 1 à 12 mois; s´exprimer verbalement en langue locale populaire (lingala) et/ou en français et être disponible pour au moins deux entretiens. Ont été exclues les mères avec handicap mental et/ou trouble de langage.

**Méthodes**: une étude transversale a été entreprise sur le vécu des mères relatif à la grossesse et à la relation avec leurs bébés, par entretiens dirigés et anamnèse sur base d´un questionnaire recueillant outre les variables sociodémographiques les données suivantes: le désir de la grossesse, les sentiments éprouvés à la connaissance de l´état de grossesse, la peur de l´accouchement, les difficultés d´accouchement, le soutien du géniteur de l´enfant, la réaction affective de la mère aux cris et ou pleurs du bébé, le délai de réponse à ces manifestations, les sensations de la mère au portage du bébé frustré, le comportement du bébé au contact d´un autre bébé et son état de santé, l´allaitement maternel. Le répertoire comportemental de Montagnier cité par Baudier et Céleste [10] a été utilisé pour évaluer le comportement social du bébé par entretien avec la mère: les offrandes et sollicitations caractérisaient le comportement affiliatif et les menaces, agressions, tentatives et actes de saisie, isolement et pleurs le comportement non affiliatif.

Les troubles affectifs de mères ont été dépistés par l´échelle de la dépression maternelle postnatale d´Edimbourg (EPDS, *Edimbourg Postnatal Depression Scale*) développée par Cox [11], les échelles de dépression et d´anxiété de Goldberg [12] ainsi que selon les critères de diagnostic clinique de la dépression du DSM-IV-R. L´EPDS a été considérée positive à la cote ≥12 points, et pour les échelles de Goldberg, le résultat était considéré positif à 2 points pour la dépression et à 5 points pour l´anxiété. Les échelles de dépression postnatale d´Edimbourg et les échelles de dépression et d´anxiété de Goldberg ont été déjà utilisés dans notre milieu [13]. Les données ont été analysées qualitativement et quantitativement, l´analyse statistique a été réalisée avec le logiciel SPSS 20,0 et recours aux tests de Khi-carré et Mann-Whitney.

## Résultats

**Caractéristiques sociodémographiques des mères et des bébés:** les enquêtées étaient âgées de 28,4 ± 12,18 ans, la plus jeune avait 14 ans et les plus âgées 44 ans. Ces mères portaient des bébés âgés d´un mois à dix mois dont 50,8% de sexe masculin (61/120). Les mères qui étaient à leur premier accouchement représentaient 34,2% des cas (41/120) et les mères comptant au moins cinq enfants 21,6% (26/120). Elles étaient ménagères dans 61,7% des cas sans autre activité rémunératrice (74/120), 16,7% s´adonnaient au petit commerce (20/120) et 21,6% à d´autres activités (26/120). Il s´agissait de femmes ayant les niveaux d´études secondaire dans 88,3% des cas (106 /120), supérieur dans 5,8% (7/120), primaire dans 1,7% (2/120); les analphabètes représentaient 4,2% (5/120). Les mères vivant une situation matrimoniale instable étaient les plus nombreuses, avec 45% d´union consensuelle (54/120); 8,3% de concubinage (10/120) et 5, 8% des mères célibataires (7/120).

**Affects de la mère pendant la grossesse:** le Tableau 1 présente les indicateurs de l´affectivité maternelle prénatale. La joie à la connaissance de l´état gravide a été éprouvée par 38,3% (46/120) comme le montre le Tableau 1. Ce tableau présente aussi les différents sentiments autres que la joie verbalisés par les enquêtées dans 61,7% des cas (74/120). Les mères ayant déclaré avoir désiré leur grossesse représentaient 37,5% des cas (45/120). Le fait de n´avoir pas désiré la grossesse étaient associé significativement à l´éprouvé des sentiments négatifs à la connaissance de l´état gravide (p<0,001): 89,1% de grossesses désirées avec sentiment de joie (41/45) et 10,9% sans joie (4/45) contre 6,7% de grossesses non désirées avec joie (5/75) et 93,3% de grossesses non désirées sans joie (70/75). La peur de l´accouchement a été rapportée dans 75,8% des cas (91/120). La grossesse non désirée était retrouvée dans 65,9% des cas de peur de l´accouchement (60/91) contre 51,7% sans cette peur (15/29) (mais p>0,05).

**Tableau 1:** indicateurs d´affectivité maternelle pendant la grossesse

Indicateurs	Fréquence	%
Désir de la grossesse	45	37,5
Sentiments à la connaissance de la grossesse		
Plus joyeuse	46	38,3
Sans joie	76	61,7
Découragée ou triste	(36)	(30)
Avec douleur au cœur	(12)	(10)
Colérique	(20)	(16,7)
Sans affects particuliers	(6)	(5)
Soutien du père de l´enfant	106	88,3
Peur de l´accouchement	91	75,8
Vécu global des événements stressants	80	66,7
Vécu des conflits conjugaux	42	35
Problèmes financiers	28	23,3
Perte d´un être cher	16	13

Le sentiment de joie à la connaissance de l´état gravide était associé à la peur de l´accouchement dans 69,6% des cas (32/46) contre 80,8% (59/73) dans les cas sans joie (p>0 ,05). La colère à la connaissance de la grossesse était associée à la peur de l´accouchement avec 85% des cas (17/20) et le sentiment de découragement/tristesse l´était avec 83,3% (30/36). Les enquêtées ont reconnu avoir eu le soutien du père de l´enfant dans 88,3% (106/120). Les mères qui s´étaient déclarées sans soutien du père de l´enfant n´avaient pas désiré leur grossesse dans 85,7% des cas (12/14), ni connu la joie à la connaissance de celle-ci dans 92,9% (13/14) et avaient eu peur de l´accouchement dans 85,7% (12/14). L´absence du soutien du père était plus associée à l´absence de joie à la connaissance de la grossesse (p=0,011): sans soutien du père et sans joie 92,8% (13/14) contre soutien du père et sans joie 57,5% (61/106); et soutien du père et joie 42,5% (45/106) contre sans soutien et joie 7,1% (1/14). Dans 66,7% des cas, les mères ont déclaré avoir connu des événements stressants dont des conflits conjugaux dans 35% (42/120), des problèmes financiers 23,3% (28/120), la perte d´un être cher 13% (16/20).

### Affects de la grossesse et problèmes de relation mère-bébé, de santé et développement du nourrisson

**Problèmes d´accouchement:** la notion de dystocie a été relevée dans 8,3% (10/120) dont 5 cas de césarienne, 2 de travail long, 1 de rétention placentaire et 2 cas d´asphyxie néonatale. La dystocie était associée dans 9 cas sur 10 à l´absence de joie à la connaissance de la grossesse et dans 8 cas sur 10 à la peur de l´accouchement.

**Relation mère-bébé, santé et développement du nourrisson:** le Tableau 2 montre que les facteurs péjoratifs étaient l´absence de joie à la connaissance de l´état gravide et l´absence du soutien du père de l´enfant pour la perception de l´état de santé de l´enfant et la dépressivité maternelle (EPDS), et la peur de l´accouchement pour l´anxiété maternelle (Echelle de Goldberg) et le comportement maternel aux signaux émotionnels du bébé.

**Tableau 2:** affects maternels pendant la grossesse et relation mère-bébé, santé et développement du bébé

Indicateurs d´affects de la grossesse	Variables associées à l´interaction mère-bébé, la santé et le développement de l´enfant	P (Tests de X^2^ et exact de Fisher)
Désir de la grossesse Vs non Désir	Dépression maternelle EPDS	0,066
	Comportement alimentaire du bébé	0,087
Différents sentiments à la connaissance de l´état gravide	état de santé de l´enfant tel perçu par la mère	0,003
	Comportement alimentaire du bébé	0,027
	Comportements maternels aux signaux émotionnels du bébé	0,030
	Réactions affectives des mères aux comportements problèmes du bébé	0,016
Joie Vs sans joie à la connaissance de l´état gravide	état de santé de l´enfant tel perçu par la mère	0,010
	Dépression maternelle EPDS	0,046
	Dépression maternelle DSM-IV	0,096
	Réactions affectives des mères aux signaux émotionnels du bébé	0,061
Absence du soutien du père de l´enfant	état de santé de l´enfant tel perçu par la mère	0,031
	Dépression maternelle EPDS	0,027
Peur de l´accouchement	Réactions affectives des mères aux signaux émotionnels du bébé	0,061
	Comportements maternels aux signaux émotionnels du bébé	0,002
	Tension chez la mère au portage du bébé frustré	0,020
	Anxiété maternelle/échelle de Goldberg	0,025

**La capacité du bébé à se détendre après frustration rapportée par la mère ou conso labilité du bébé:** les cas de bébés qui se calmaient facilement après frustration représentaient 79,2% (95/120), ceux difficiles à calmer 17, 5% (21/120) et ceux à comportement variable 3,3% (4/120). Au regard du Tableau 3, les cas des bébés se détendant difficilement avaient une histoire de grossesse sans joie dans 71,4% (15/21), grossesse non désirée avec découragement/tristesse 100% (13/13) et mères à sentiments douloureux aux cris/ pleurs du bébé dans 80,9% (17/21). Les 4 cas de comportement variable avaient tous une histoire de peur de l´accouchement et de mères à sentiments douloureux dans 3 cas sur 4. Les cas faciles à calmer étaient associés à la peur de l´accouchement dans 68,7% (65/95) et à la douleur chez les mères dans 68,4% (65/95).

**Tableau 3:** histoire affective de la grossesse et profil de consolabilité du bébé (comportement du bébé après frustration)

Sentiments à la connaissance de l´état gravide	Comportements de consolabilité du bébé	Fréquence	% P
Joie (n=46)	Détente facile	39	84,8
	Détente difficile	6	13
	Comportement variable	1	2,2
Colère (n=20)	Détente facile idth=97 valign=top> 18	90
	Détente difficile	2	10
Douleur au cœur (n=12)	Détente facile	8	66,7
a	Détente difficile	4	33,3
Tristesse/découragement (n=36)	Détente facile)	27	75
	Détente difficile	8	22,2
	Comportement variable	1	2,8
Sans affects particuliers (n=6)	Détente facile	3	50
	Comportement variable	2.	33,3
	Détente difficile	1	16,7 p=0,004
Détente difficile (n=21)	Grossesse non désirée et absence de joie (découragement/tristesse)(n=13)	13	100
	Grossesse désirée et joie (n=8)	6	75
	Grossesse désirée et absence de joie (n=8)	2	25 p<0,001
Détente facile (n=95)	Grossesse désirée et joie (n=35)	34	97,1
	Grossesse désirée sans joie (n=35)	1	3,8
	Grossesse non désirée sans joie (n=60)	55	91,7
	Grossesse non désirée avec joie (n=60)	5	8,3 p<0,001

### Histoire affective de la grossesse et relation mère-bébé

**Réactions affectives et comportementales de la mère aux cris et pleurs du bébé:** la douleur au cœur a été verbalisée dans 69,1% des cas (85/120), la colère dans 10% (12/120), le découragement/ sentiment d´impuissance dans 10% et l´absence d´affects particuliers et autres sentiments dans 9,1% (11/120). La colère, dans 91,7% (11/12), était associée à l´absence de joie à la connaissance de la grossesse, dans 83,3% des cas (10/12) à la grossesse non désirée et dans 83,3% (10/12) à la peur de l´accouchement. L´absence d´affects particuliers faisait suite à la peur de l´accouchement dans 80% (4/5). La douleur au cœur était associée à la peur de l´accouchement dans 78,8% (67/85). Les comportements maternels aux signaux émotionnels de bébés ont montré que l´absence de réponses dans 5 cas sur 6 était en continuité avec l´absence de joie à la connaissance de la grossesse. Dans ce cas d´absence de joie, l´absence du soutien du père était un facteur favorable aux réponses maternelles retardées: réponses retardées dans 9 cas sur 9 en cas d´absence combinée de joie et de soutien du père et dans 55,1% (38/69) dans l´absence de joie associée au soutien du père versus 44,9% (31/69) dans la combinaison joie et soutien du père de l´enfant (p=0,010). L´ennui à porter le bébé frustré faisait suite à une histoire de grossesse non désirée et d´absence de joie dans 3 cas sur 4, et à une histoire de peur de l´accouchement dans un 1 cas sur 4. La peur de l´accouchement était associée à 13 cas de tension corporelle chez la mère au portage du bébé frustré sur 14 (92,9%) contre 75 cas d´absence de tension sur 102 (73,5%) et 1 sur 4 cas d´ennui au portage du bébé frustré (p=0,02).

**Allaitement maternel exclusif:** l´allaitement maternel exclusif dans les 6 premiers mois a été retrouvée dans 90% (108/120) contre 10% (12/120) d´allaitement non exclusif. Ces derniers concernaient des bébés nés de grossesses non désirées avec notion d´absence de joie à la connaissance de la grossesse chez la mère dans 66,7% (8/12) *versus* 33,3% (4/12) de grossesse désirée avec sentiment de joie (p=0,001).

**Dépressivité et dépression maternelles:** l´anxiété à l´échelle de Goldberg était observée dans 62,3% (33/53) de cas de dépression à l´échelle de Goldberg, 60% (37/61) à l´échelle de dépression postnatale d´Edimbourg et 70,9% (22/31) au DSM-IV (p<0,001 dans les 3 situations). L´anxiété était en lien dans 40% (37/91) de cas avec la peur d´accouchement contre 17,2% (5/29) sans peur de l´accouchement (p=0,021). La fréquence de l´anxiété était relativement plus basse dans les cas de réponses maternelles retardées que dans les réponses immédiates, avec des scores plus élevés dans ces derniers que dans les premiers (p=0,015). Le Tableau 4 présente des facteurs interactionnels associés à des fréquences et scores élevés de dépression. Il s´agit de la peur de l´accouchement, du manque du soutien du père, de l´état de santé et du comportement non affiliatif du bébé. Le taux d´anxiété maternelle dans les cas d´état de santé moins bon était de 52% (13/25) contre 30,5% (29/95) dans le cas d´état de santé bon (p=0,045).

**Tableau 4:** facteurs associés à des scores élevés aux échelles de dépressivité maternelle

Variables et Mesures		Comparaison des rangs moyens des scores (Test de Mann-Whitney)	
		EPDS	échelle de dépression de Goldberg
		Rangs moyens; p	Rangs moyens; p
Désir d´une naissance	Grossesse non désirée vs Grossesse désirée	65,01 vs 52,99; p=0,066	65,24 vs 52,60; p=0,051
Sentiments à la connaissance de la grossesse	Découragement/tristesse vs Joie	47,88 vs 36, 51; p=0,032	48,49 vs 36,03; p=0,017
	Sans joie vs Joie	65,97 vs 51,70; p=0,028	66,79 vs 50,38; p=0,011
Peur accouchement	Peur vs Absence de peur	64, 04 vs 49,40; p=0,048	64,27 vs 48,67; p=0,033
Soutien du père de l´enfant	Non vs Oui	80,00 vs 57,92; p=0,025	79, 50 vs 57,94; p=0,027
Stress pendant la grossesse	Conflits conjugaux vs Perte d´être cher	29,16 vs 17,72; p=0,009	28,72 vs 18,66; p=0,021
Etat santé de l´enfant perçu par la mère	Moins bon vs Bon	75,74 vs 56,49; p=0,014	75,46 vs 55,51; p=0 ,002
Bébé au contact d´autres bébés	Menace, attaque vs Sollicite, approche	33,17 vs 24 ,28; p=0,038	34,52 vs 24, 30; p=0 ,018
Anxiété à l´échelle de Goldberg	Anxiété positive vs Anxiété négative	86,30 vs 46,61; p=0,001	83,57 vs 48,08; p=0,001

**Comportement social du bébé envers d´autres bébés perçu par la mère:** les stéréotypes suivants ont été identifiés avec les mères (n=65): 52,3% des cas (34/65); le comportement agressif ou non affiliatif (38,5% (24/65) et d´autres comportements dans 9,2% (7/65). Le comportement affiliatif était observé à la même fréquence de 50% (17/34) dans les 2 sexes alors que d´autres modalités ont concerné le garçon dans 54,8% (17/31) contre la fille 45,2% (14/31) (p>0,05). L´association grossesse désirée-absence de joie à la connaissance de l´état gravide dans 3 cas sur 3 donnait lieu au comportement non affiliatif chez le bébé contre 7 sur 20 de comportement non affiliatif (35%) et 13 sur 20 (65%) de comportement affiliatif dans les suites de l´association grossesse désirée-joie(p=0,034). La dépression maternelle postnatale à l´EPDS faisant suite à la grossesse désirée (n=23) était plus associée au comportement non affiliatif chez le bébé, 75% dans les cas de dépressions (6/8) contre 33,3% (5/15) sans dépression (p=0,026).

**Perception de l´état de santé du bébé par la mère et l´affectivité maternelle pré et postnatale:** les mères avaient apprécié l´état de santé de leurs bébés “bon” dans 79,2% (95/120) et “moins bon” 20,8% (25/120). L´appréciation bonne était retrouvée dans la suite du sentiment de joie à la connaissance de la grossesse dans 91,3% (42/46) vs 71,6% (53/74) dans l´absence de joie. L´appréciation moins bonne concernait 28,4% des cas d´absence de sentiment de joie (21/74) vs 8,7% des cas de sentiment de joie(4/46) (p=0,010). Le soutien du père de l´enfant était plus associé aux cas d´état de santé de l´enfant apprécié bon dans 91,6% (87/95) contre 76% (19/25) d´état de santé apprécié moins bon, 8,4% (6/95) d´état de santé bon et 31,6% (6/19) de moins bons sans soutien du père (p=0,031; OR=3,4; IC 95%: 1,1-11,1). L´appréciation moins bonne de l´état de l´enfant par la mère tendait à s´associer à l´histoire de la peur de l´accouchement 80% (20/25) contre 74,7% (71/95) en cas de bonne santé.

## Discussion

**Sentiments de la mère pendant la grossesse, affectivité maternelle prénatale et postnatale:** les résultats de notre étude montrent que les sentiments de la mère relatifs à la grossesse et l´accouchement constituent des indicateurs pertinents du désir de grossesse et de l´affectivité maternelle prénatale et postnatale. L´affectivité est comprise ici comme “capacité individuelle à éprouver des sentiments ou des émotions et la réaction émotive généralisée ayant des effets définis sur le corps et l´esprit ” et comme “ensemble de comportements permettant les rapports sociaux individuels intimes qui lient entre eux les membres d´une même espèce” [10]. L´affectivité maternelle comprend l´ensemble des affects, émotions et sentiments éprouvés par la mère et sous-tendant de façon globale ses comportements et ses pensées dans la relation avec le bébé. L´affectivité assure la continuité et la cohérence de l´expérience en dépit de changements du milieu et constitue le fondement de la communication et de l´interaction interpersonnelle [14, 15]. Cette continuité est illustrée dans la présente étude par l´association entre l´absence de joie à la connaissance de la grossesse et les affects et comportements négatifs de la mère avant l´accouchement et après la naissance. Ces processus psycho-affectifs de la maternité inscrivent l´histoire prénatale dans le développement de l´enfant [16-18]. L´étude des sentiments de la mère pendant la grossesse, une particularité de notre travail, montre que le processus d´interaction interpersonnelle amorcé par les premiers sentiments de la mère à la connaissance de son état gravide constitue un déterminant psychosocial central de l´affectivité maternelle et du développement du bébé, cela en accord avec les données de la littérature sur la psychopathologie périnatale [18-21].

Les sentiments négatifs à la connaissance de l´état gravide portent une empreinte négative sur la relation mère/bébé, la santé et le développement du bébé. Cela peut s´expliquer par les troubles de l´investissement émotionnel du bébé et leurs conséquences décrites par Figueiredo *et al*. [18] et par les effets délétères durables du stress maternel prénatal relatif à la grossesse non désirée sur le développement mental de l´enfant et la santé mentale de la femme [6, 22]. Une grossesse non désirée peut être vécue comme une crise psychique, un échec personnel avec atteinte de l´estime de soi et cause de discordance interpersonnelle [23-25], autant de facteurs de la dépression et de troubles de l´affection maternelles par atteinte narcissique. Selon Louis, la grossesse non désirée est un facteur de risque de dépression maternelle postnatale indépendant et donnant des scores élevés à l´échelle de dépression postnatale d´Edimbourg [26]. Notre étude met plus en évidence le rôle des sentiments négatifs de la mère associés à l´état gravide qui élevaient la fréquence et surtout les scores de dépression à l´EPDS. L´expression des affects négatifs relatifs à la grossesse constitue ainsi un indicateur pertinent de la grossesse non désirée, un marqueur négatif de l´affectivité maternelle pré et postnatale.

La perception négative de la grossesse est considérée comme le seul critère spécifique de la dépression maternelle prénatale [20]; elle constitue un facteur de risque de la dépression maternelle postnatale justifiant 10 à 60% des cas selon les études citées par Dayan *et al*. [20, 21]. L´attitude de la mère au cours de la grossesse à l´égard de son mariage et de l´enfant s´avère un bon prédicteur de dépressions maternelles postnatales [19]. Les fréquences élevées des affects négatifs relatifs à la grossesse, l´accouchement et la relation mère-bébé et de la dépression maternelle postnatale dans notre étude témoigneraient aussi de la détresse maternelle associée à une pauvreté chronique [27] et des séquelles des violences que subissent la femme et la jeune fille dans notre milieu au regard des données de l´OMS sur les conséquences des violences [28]. Les femmes qui ont des grossesses non désirées ont plus de troubles psychologiques et sociaux et vice-versa [22, 29, 30].

**Affectivité maternelle prénatale et postnatale, relation mère-bébé, santé et développement du bébé:** dans notre étude, de plus nombreuses histoires d´absence de joie à la connaissance de l´état gravide, associées à la grossesse non désirée, sont en rapport avec les réponses maternelles négatives comme la colère aux cris et pleurs du bébé, le retard ou absence de réponse aux signaux émotionnels du bébé, l´ennui au portage du bébé frustré, l´allaitement maternel non exclusif dans les six premiers mois et les problèmes de santé et développement du bébé en périodes périnatale et postnatale. En effet, les soins prénatals, l´accouchement et les consultations préscolaires sont décrits mieux réalisés dans les suites d´une grossesse désirée [29]. Le stress maternel pendant la grossesse a des effets négatifs sur le développement prénatal et postnatal de l´enfant, notamment sur le registre moteur et le contrôle du comportement, surtout en conditions de vie défavorables [4, 31, 32]. Dans notre étude, le lien des affects négatifs de la grossesse avec la conso labilité du bébé de même qu´avec son comportement non affiliatif rend compte, entre autres, du rôle du stress maternel prénatal dans les troubles émotionnels et cognitifs de l´enfant [33]. L´association de la peur de l´accouchement à des fréquences élevées d´anxiété maternelle postnatale, de difficultés à se détendre au portage du bébé frustré ou à répondre adéquatement aux signaux émotionnels du bébé et à ses problèmes de santé montre que cette crainte peut être liée à la personnalité de la mère ou au stress maternel passé ou actuel. Toutefois, une plus grande présence de symptômes anxieux pendant la grossesse fait prévoir plus d´émotions négatives à l´égard du bébé [18].

La dépression maternelle postnatale était relation avec le mauvais état de santé du bébé rapporté par la mère. La littérature rapporte les préoccupations des mères déprimées sur la santé de leurs bébés, l´effet dépressif des mauvais états de santé de bébés sur les mères [10, 19] ainsi que les effets durables du stress maternel prénatal sur la santé et le développement du bébé [3, 6]. Les données de notre étude peuvent servir d´indicateurs pour le repérage et la surveillance cli niques dès le début de la grossesse des troubles de l´affectivité maternelle, de la relation mère-bébé et développementaux de l´enfant en soins de santé primaires. Dès la naissance, les dysfonctionnements moteurs et le tempérament difficile du bébé augmentent le risque de dépression chez la mère [34, 35] alors que le développement neurologique pathologique et le handicap du nourrisson font de même [30, 36]. Les effets du stress maternel pendant la grossesse ont été déjà décrits en termes des troubles neurologiques et des retards développementaux chez l’enfant [37]. Les troubles du développement sont attribuables aux modifications durables du fonctionnement de l´axe hypothalamo-hypophyso-cortico-surrénalienne, du dispositif perceptuel congénital et du système des morphines endogènes [1, 3, 38, 39], l´hyperactivité adrénergique et l´hypercortisolémie associées à la détresse psychologique maternelle avec anxiété prédominante [39, 40, 41]. La structure et de la fonctionnalité de l´hippocampe, de noyau amygdalien et du cortex préfrontal sont affectées par l´effet des minéral corticoïdes et des glucocorticoïdes [1, 33, 42]. L´ensemble des données précitées et propres à notre étude appuient l´hypothèse de notre travail concernant les effets péjoratifs du stress associé aux sentiments négatifs de la future mère contemporains à la grossesse.

**Les sentiments de la mère pendant la grossesse, typologie affective des mères et la neuroprotection psychosociale:** les résultats de notre étude montrent le rôle des sentiments de la mère à la connaissance de l´état gravide dans le fondement affectif de la relation mère-enfant et la caractérisation des mères en rapport avec le stress prénatal et postnatal associé à la grossesse non désirée. Les mères en situation de naissance non désirée présentent une plus grande hostilité et des fantasmes de destruction envers le fœtus, un défaut d´affection maternelle avec risque de néonaticide, moins de sentiments positifs, des sentiments négatifs accrus et un lien moins étroit avec l´enfant [20, 21, 26, 43]. Mais les facteurs de personnalité de la mère, non étudiés dans cette étude, peuvent de manière pertinente être le fondement du maintien durable et stable d´une relation sécurisante mère-enfant [44]. Ces facteurs opèrent de façon permanente. Dans des travaux ultérieurs, les caractéristiques affectives et comportementales de la mère seront étudiées en lien avec son tempérament et sa personnalité, l´histoire de la grossesse et le stress maternel prénatal et postnatal, le développement physique et neurologique et le tempérament du bébé. Ces études sont utiles pour l´élucidation de l´impact du stress prénatal dans la psychopathologie maternelle, la caractérisation des mères et la neuropsychopathologie du bébé.

## Conclusion

Les affects négatifs relatifs à la grossesse et l´accouchement constituent des marqueurs pertinents de la dépression maternelle, de troubles de la relation mère/bébé et de problèmes de santé et du développement du nourrisson. Ce risque est associé à la grossesse non désirée et au stress maternel prénatal. Les affects de la mère pendant la grossesse et en relation avec l´accouchement sont à prendre en compte en clinique et dans la recherche de l´impact du stress prénatal dans la psychopathologie maternelle et la neuro-psychopathologie du bébé. Il en découlerait une perspective de la neuroprotection psychosociale dans le cadre de la santé maternelle et infantile en soins de santé primaires et conditions de vie défavorables.

### Etat des connaissances sur le sujet

- La grossesse non désirée et l´affectivité maternelle prénatale, comme déterminant psychosocial de la santé et développement de l´enfant, sont difficiles à mesurer;
- Le stress prénatal, notamment celui de la grossesse non désirée et les troubles affectifs pendant la grossesse sont associés à des problèmes de santé et de développement en période périnatale et tout au long de la vie;
- La recherche pour la prévention efficace de ces problèmes nécessite une approche diagnostique, clinique et des interventions individualisées, cette approche fait figure encore de parent pauvre en soins de santé maternelle et infantile primaires.

### Contribution de notre étude à la connaissance

- En soins de santé primaires, dans le cadre de consultations préscolaires, cette étude met en évidence la continuité entre l´affectivité maternelle prénatale, le stress émotionnel postnatal dans la relation mère-enfant et la dépressivité maternelle postnatale;
- Elle montre les liens entre les sentiments négatifs de la mère relatifs ou contemporains à la grossesse et les problèmes d´accouchement, santé et comportement du nourrisson;
- L´étude clinique de ces sentiments montre qu´ils constituent un indicateur pertinent de la grossesse non désirée, un marqueur valide de l´affectivité maternelle pré et postnatale, de la relation mère-enfant et de la santé et développement psychosocial précoce.

## Remerciements

Les auteurs remercient les responsables du Centre Hospitalier de Kingasani de la Ville de Kinshasa (RDC) qui ont accepté et encouragé la réalisation de cette étude et offert le cadre de travail, les mères et leurs bébés à Kinshasa dont la situation sociale avaient inspiré cette étude et qui avaient consenti d´y participer. Ils remercient également le Professeur Désiré Tshala Katumbayi pour ses précieux conseils et encouragements pour la rédaction de ce travail.
